# Measles

**Published:** 1878-04-20

**Authors:** 


### MEASLES.

The measles is extensively prevailing in this vicinity. Many say that they can't tell where they got it unless they "*caught it at the circus.*" We are satisfied that infectious diseases, especially measles and scarlatina, are transmitted from point to point and scattered broadcast by these traveling companies. It is affirmed, however, of measles, that the gathering together of multitudes, or the mingling of people of different races, or from different neighborhoods or classes of society, will generate the disease. Hence, in times of war, when men go into camp, rubeola is usually the first disease that makes its appearance.

---

BANDANNAH.—We have good reason to believe that in admitting the *bandannah* advertisement to our pages we were imposed upon, and that it is a humbug. Such things are liable to happen occasionally, despite the greatest care. The best we can do in such cases is to take it out at once and place our readers upon their guard, which we have done.

---

LUDDEN & BATES, the great piano men of the South, have another advertisement in the present number of our journal. We commend them to any who may desire to purchase pianos or organs.

